# Liver Dysfunction in a Patient with Graves’ Disease

**DOI:** 10.3390/jcm13226968

**Published:** 2024-11-19

**Authors:** Filipa Campos, Angelica Sharma, Bijal Patel, Deborah Papadopoulou, Alexander N. Comninos, Ali Abbara

**Affiliations:** 1 Section of Endocrinology and Investigative Medicine, Imperial College London, London W12 ONN, UK; 2 Division of Endocrinology, Imperial College Healthcare NHS Trust, London W12 ONN, UK

**Keywords:** hyperthyroidism, carbimazole, Grave’s disease, hepatitis, liver dysfunction

## Abstract

Liver dysfunction can occur in patients presenting with thyrotoxicosis, due to several different aetiologies. A 42-year-old man had mild liver dysfunction on presentation with hyperthyroidism due to Graves’ disease (GD): ALT 65 (0–45 IU/L), fT4 41.2 (9–23 pmol/L), fT3 > 30.7 (2.4–6 pmol/L), and TSH < 0.01 (0.3–4.2 mIU/L). His liver dysfunction worsened following the initiation of the antithyroid drug (ATD) carbimazole (CBZ), with ALT reaching a zenith of 263 IU/L at 8 weeks following presentation. Consequently, CBZ was stopped, and he was managed with urgent radioiodine therapy. His liver function tests (LFTs) improved within 1 week of stopping carbimazole (ALT 74 IU/L). Thionamide-induced liver dysfunction is more typically associated with a ‘cholestatic’ pattern, although he had a ‘hepatitic’ pattern of liver dysfunction. The risk of liver dysfunction in GD increases with older age and higher titres of thyroid-stimulating hormone receptor antibody (TRAb). This review of the literature seeks to explore the possible causes of liver dysfunction in a patient presenting with hyperthyroidism, including thyrotoxicosis-induced liver dysfunction, ATD-related liver dysfunction, and the exacerbation of underlying unrelated liver disease.

## 1. Introduction

Biochemical liver derangement is found in 15–75% of patients with untreated hyperthyroidism [1,2]. Whilst mostly asymptomatic, 1–2% can manifest acute hepatitis and liver failure [3]. Key causes of liver dysfunction in patients with hyperthyroidism include direct effects of thyrotoxicosis [4]; the effects of autoimmune Graves’ disease (GD); side effects of ATDs (e.g., thionamides); or incidental non-thyroid-related causes of liver disease, e.g., metabolic dysfunction-associated steatotic liver disease (MASLD) or autoimmune liver disease [2]. In practice, managing patients with hyperthyroidism and coexistent hepatic dysfunction represents a relatively common clinical scenario but can be difficult to easily identify the specific cause of liver dysfunction to determine optimal management. In particular, it can be challenging to determine whether liver dysfunction is a direct result of thyrotoxicosis, in which antithyroid drugs (ATDs) can be continued and liver dysfunction should improve as the thyrotoxicosis is resolved, or if the liver dysfunction is attributable to the ATDs themselves, necessitating the consideration of alternative treatment modalities. Additionally, the autoimmune dysfunction associated with GD can also cause liver dysfunction.

This case report and discussion explore the clinical course of a man presenting with hyperthyroidism due to GD with concomitant liver dysfunction that worsened following the commencement of ATD. We provide an in-depth discussion on decisions regarding his management and review the literature to summarise the characteristic features of different causes of liver dysfunction in patients with GD.

## 2. Case Presentation

A 42-year-old man presented with a three-month history of weight loss (~10–11 kg), intermittent palpitations, peripheral tremors, increased bowel movement frequency (4× per day), and mild abdominal cramps. He did not have fever, pain, dyspnoea, dizziness, dysuria, urinary urgency, or frequency. He had no visual symptoms associated with GD. He had not experienced any preceding coryzal symptoms, intercurrent flu-like illness, fever, or night sweats. He had no past medical history, nor took any medications. He had a strong family history of GD, with both his mother and brother having been diagnosed with GD. He was a non-smoker but reported regular (and likely significant) alcohol intake (~24 units per week). He worked in banking and was married with a young daughter.

## 3. Clinical Findings

### On Examination

On initial presentation, he was tachycardic (regular pulse rate of 116 bpm), afebrile (temperature: 36.9 °C), blood pressure 125/80 mmHg, normal oxygen saturations on room air of 99% and respiratory rate (16 bpm), with normal body habitus (weight 78.3 kg).

He appeared well with a fine tremor, and a small, painless goitre. He had mild exophthalmos but no other abnormalities on ophthalmological examination. Cardio-respiratory examination was normal. The abdomen was non-distended, soft, and non-tender. His calves were soft and non-tender, with no peripheral oedema, nor any evidence of pre-tibial myxoedema.

## 4. Investigations

An electrocardiogram (ECG) showed sinus tachycardia. Other blood investigations showed haemoglobin 117 (130–180 g/L), MCV 69.4 (80–100 fL), normal white cell count (5 × 10^9^/L (3.6–11 × 10^9^/L)), and C-reactive protein 1.3 (<5 mg/L). Thyroid function tests (TFTs) revealed an undetectable TSH < 0.01 (0.3–4.2 mIU/L), raised free thyroxine (fT4) 41.2 (9–23 pmol/L), free triiodothyronine (fT3) > 30.7 (2.4–6.0 pmol/L)), and raised thyroid-stimulating hormone receptor antibody (TRAb) titre 10 (0.0–0.9 IU/L) consistent with hyperthyroidism due to GD. At presentation, he had a mildly raised ALT of 65 (0–45 IU/L) but an ALP within the normal limits of 85 (30–130 µmol/L). The technetium 99 m pertechnetate scan showed diffuse bilateral increased uptake (10.2%).

## 5. Initial Management

In view of his clinical, biochemical, and radiological findings of hyperthyroidism, he was formally diagnosed with hyperthyroidism due to GD and was commenced on CBZ 40 mg once daily (OD) and propranolol 10 mg OD.

## 6. Follow-Up and Outcomes

**Figure 1:**
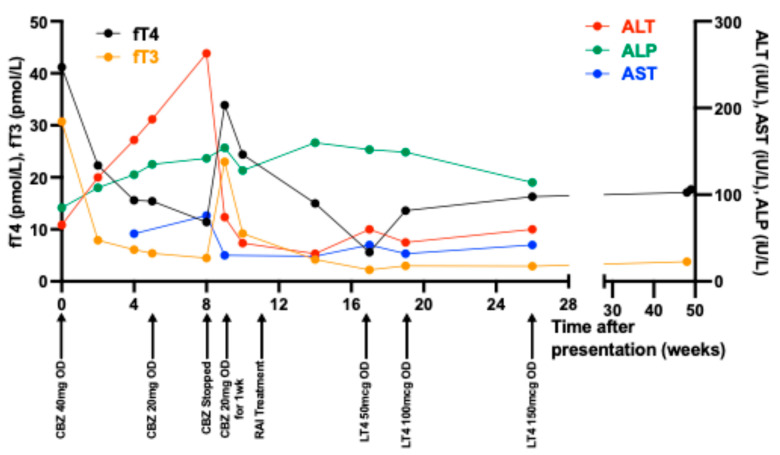
The trajectory of thyroid function tests and liver function tests with treatment. Abbreviations: fT4, free thyroxine; fT3, free triiodothyronine; ALT, alanine aminotransferase; AST, aspartate aminotransferase; ALP, alkaline phosphatase; CBZ, carbimazole; RAI, radioiodine; LT4, levothyroxine.

He was diagnosed with hyperthyroidism due to GD and commenced on CBZ 40 mg once daily (OD) and propranolol 10 mg OD. Upon review at 2 weeks following starting CBZ, his TFTs had improved, with TSH < 0.01 (0.3–4.2 mIU/L), fT3 7.9 (2.4–6.0 pmol/L), and fT4 22.3 (9–23 pmol/L); however, his LFTs had worsened, with ALT rising to 120 (0–45 IU/L) from 65 IU/L (Table 1). His ALT continued to rise to 163 (0–45 IU/L) at 4 weeks (Figure 1) and further to 187 IU/L (0–45 IU/L) at 5 weeks. Therefore, his dose of CBZ was reduced from 40 mg to 20 mg OD (Figure 1). Nonetheless, his LFTs continued to worsen, peaking 3 weeks later with ALT 263 (0–45 IU/L), AST 76 (0–40 IU/L), and ALP 142 (30–130 µmol/L), and bilirubin 11 (0–21 µmol/L)), consistent with a ‘hepatitic’ picture. A full liver screen including viral and autoimmune serology and a liver ultrasound scan found only mild hepatic steatosis.

Given his worsening liver dysfunction, his CBZ was stopped, and he was managed with urgent RAI 3 weeks later. Within two weeks after RAI, his fT4 and fT3 normalised and his LFTs also normalised, aside from a mildly raised ALP 160 (30–130 µmol/L) (Table 1). At 6 weeks after RAI, the patient developed hypothyroidism and was commenced on levothyroxine and adjusted until euthyroid (Table 1).

Due to improvements in his liver function tests, a liver biopsy was deemed unnecessary. Consequently, a limitation of this case is the absence of histopathological findings to corroborate the abnormalities in LFTs.

## 7. Discussion and Review of Literature

### 7.1. Graves’ Disease Induced Hepatic Dysfunction

Hepatic dysfunction in patients with GD ranges from subclinical biochemical abnormality to acute hepatitis [5]. Several mechanisms can contribute to liver dysfunction in the context of GD [4]. The complex interaction between the thyroid gland and the liver is crucial for maintaining physiological homeostasis [5]. In patients with GD, 58.5% develop mild hepatic dysfunction (ALP < 2× upper limit of normal (ULN), ALT or AST or GGT < 3× ULN, or Bn < 2.5× ULN), 34.9% moderate (ALT or AST 3–20× ULN, or GGT 3–10× ULN, ALP 2–5× ULN, or Bn 2.5–5× ULN), and 6.6% severe (ALT or AST ≥ 20× ULN, GGT ≥ 10× ULN, ALP ≥ 5× ULN, or Bn ≥ 5× ULN) [6]. Hepatic dysfunction was further classified as hepatitis in 45.8%, biliary stasis in 32.4%, and mixed in 21.8% [6]. Abnormal ALP is present in 44–52.3% [6], ALT in 33%, AST in 23%, bilirubin in 12%, and GGT in 24% [2].

In GD, TRAb can also bind to TSHR on hepatocytes, leading to inflammatory injury [2,7], which could contribute to the increased incidence of liver dysfunction in GD compared to other types of thyrotoxicosis. In a meta-analysis [2] of newly diagnosed untreated thyrotoxicosis, 50% had at least one LFT abnormality, as compared with 66% of patients with GD. Although GD is the most common form of hyperthyroidism, many studies reviewing liver dysfunction in hyperthyroidism do not differentiate the presentation by the cause of thyrotoxicosis [8].

### 7.2. Thyrotoxicosis-Induced Liver Dysfunction

Another possible cause of liver dysfunction is an increase in metabolic activity associated with thyrotoxicosis [3]. Deiodinases in the liver play a key role in regulating the activity of thyroid hormones. Around 80% of T3 is synthesised via the 5’-iodination of T4 in the liver and kidney [3,9]. Additionally, >99% of thyroid hormones are bound to proteins produced in the liver [3,10]. Thyrotoxicosis results in higher oxygen demands on the liver but without a concomitant increase in hepatic blood flow, occasionally resulting in hepatocyte ischaemia and infarction [1,3]. This may be reflected by transaminitis, as in this case. Less is understood about the mechanisms behind thyrotoxicosis-induced cholestasis; however, increased oxygen demands in the liver causing hypoxia in the centrilobular zones of the liver may interfere with the transport of bile and cause cholestasis [1,11,12,13]. Liver biopsy specimens can show intracanalicular cholestasis and mononuclear cell infiltrates in the portal triad of the liver lobules [14].

### 7.3. ATD-Induced Liver Dysfunction

Upon diagnosis of GD, CBZ or PTU are the most commonly used first-line medications in the United Kingdom [15]. Overall, the incidence of liver dysfunction due to ATDs is estimated at 0.1–0.2% [16]. Classically, PTU results in hepatocellular toxicity with liver biopsy demonstrating nonspecific hepatocellular necrosis [17], whereas CBZ or methimazole (CBZ is a prodrug, whereas methimazole is its active metabolite) more typically results in cholestatic jaundice without hepatic necrosis [18]. In 37,370 patients treated with CBZ, cholestasis occurred at 0.24 per 1000 person-years [19]. There was a higher incidence of cholestatic injury in patients taking CBZ/methimazole (35.3%) than PTU (17.9%) [20].

CBZ is metabolised by cytochrome P450 (CYP450) enzymes, which can produce reactive intermediates such as N-methylthiourea and glyoxal, which have been proposed as cytotoxic culprits for liver damage [21]. Most CBZ-induced hepatitis reports show histological changes consistent with mononuclear cell infiltration in biliary ducts [14]. LFTs in this case displayed a hepatitic picture rather than the more usually encountered cholestatic picture.

The underlying mechanism of liver injury secondary to PTU is less clear. There are no reports of direct toxic metabolites causing hepatic damage; however, PTU is metabolised in the liver by glucuronidation and may inhibit enzymes that play a role in defence against toxins, hence exposing hepatic cells to injury [21].

Asymptomatic increases in AST/ALT occur in ~30% of patients treated with PTU [4,22]. Increases of up to 3× ULN in LFT levels are seen in ~4% of patients treated with PTU [23], which is greater than other thionamides. The rise in LFTs appears dose-dependent, and their levels are highest within the first few weeks of treatment, rapidly declining following dose reduction [24]. This reaction to PTU most commonly occurs within the first 2–3 months of treatment [2], at a median of 120 days post-initiation [25].

Liver dysfunction is less common with CBZ (incidence of 0.1–0.2%) [16], with only a few documented cases describing mild cholestatic liver injury [26]. CBZ or methimazole-induced hepatotoxicity usually occurs more rapidly than PTU, as early as 2 weeks after starting the drug [27]. Although there is a faster onset of LFT derangement in patients taking CBZ (2–3 weeks) as compared to those on PTU (2–3 months), elevations in liver enzymes with CBZ can persist for several months after stopping [27]. LFTs usually resolve without intervention between 16 to 145 days following PTU cessation [28].

### 7.4. Risk Stratification of Liver Dysfunction

Age > 45 years, history of GD > 3 years, heart rate > 90 bpm, fT3 > 3× ULN, TRAb > 10× ULN, and positive thyroid peroxidase antibody (TPOAb) were more likely associated with LFT derangement [6]. Increased age, hyperthyroidism duration, heart rate, thyroid weight, fT4, radioiodine uptake of thyroid, thyroglobulin antibody, TPOAb, and TRAb were the most significant risk factors for developing hepatic injury in patients with hyperthyroidism [7]. There was a significant correlation between TRAb and AST, ALP, GGT, and bilirubin, but not ALT [7].

### 7.5. Treatment Guidelines

Some guidelines recommend routine monitoring of LFTs in all patients within 3 months of commencing drugs [24]. In the United Kingdom, the National Institute for Health and Care Excellence (NICE) guidelines recommend that LFTs are checked before starting ATDs but advise against routine monitoring unless there is clinical suspicion [15]. In the United States of America, American Thyroid Association (ATA) guidelines also recommend only measuring LFTs in patients who experience symptoms suggestive of liver dysfunction, including a pruritic rash, jaundice, dark urine or light-coloured stools, joint pain, abdominal pain, anorexia, nausea, or fatigue [27].

PTU can cause fatal hepatic necrosis seen on liver biopsy in approximately 0.1% of patients [28], in some cases requiring liver transplantation, which led to recommendations that patients taking this particular ATD should have routine monitoring of LFTs, especially throughout the first 6 months of therapy [27]. ATA guidelines [27] state that routine LFT monitoring in patients taking thionamides can prevent severe liver derangement; however, if carried out, ideally should occur within 120 days of ATD initiation when most cases of hepatotoxicity occur. ATA guidelines [27] recommend stopping thionamides if transaminases are >5× ULN. Once an ATD is stopped, LFTs should be monitored weekly until normalisation. If the resolution of LFTs does not occur, a hepatology review is indicated [27].

The normalisation of LFTs with the return of euthyroidism occurs in 77–83% of patients with baseline abnormal LFTs treated with ATDs [3,29], with the time to normalisation after achieving euthyroidism ranging between 6 weeks and 12 months. The frequency of normalisation of specific LFTs was as follows: ALT in 83%, AST in 87%, ALP in 53%, bilirubin in 50%, and GGT in 70% [2].

### 7.6. Other Potential Causes of Liver Dysfunction

GD may also occur in conjunction with other autoimmune conditions, such as primary biliary cirrhosis, autoimmune cholangiopathy, and autoimmune hepatitis [30]. The co-occurrence of GD autoimmune hepatitis occurs in 6–10% of patients [4].

Thyroid hormone exerts effects on osteoblasts to stimulate osteoclastic bone resorption [31]. Elevated ALP levels may persist for several months following the resolution of thyrotoxicosis as increased bone turnover likely persists for longer [31]. Thus, the measurement of bone-specific ALP and liver-specific ALP could aid in confirming the source of isolated raised ALP.

This gentleman also had a fatty liver on ultrasound and increased alcohol intake. However, on a review of hepatology, it was felt that he had a low risk of fibrosis, and the likeliest cause was hepatic dysfunction secondary to the ATD given the rapid resolution following the cessation of the ATD. He was screened for other causes of liver dysfunction including autoimmune causes, but these were all normal. Propranolol is not thought to commonly cause liver dysfunction and is unlikely to have contributed adversely to his presentation.

### 7.7. Challenges in Clinical Practice

It is challenging to predict if and when liver dysfunction occurs following the introduction of ATD therapy. Without pre-existing LFTs to determine the time course of dysfunction, it is also difficult to determine whether the liver derangement is a result of thionamides or thyrotoxicosis itself, autoimmune thyroid disease, or due to incidental unrelated causes of liver disease that were already present or exacerbated. There is a lack of consensus on the optimal interval of liver function monitoring in individuals commenced on ATD medications. We noted a rise in transaminases approximately 2 weeks following the introduction of CBZ, which coincides with the literature (on average 2–3 weeks after starting the medication [18,26]. In the United Kingdom, NICE guidelines [15] suggest that LFTs should be assessed within 3 months of starting thionamides; however, they advise against routine monitoring throughout the duration of treatment. The early identification of transaminitis and stopping ATD treatment results in the reversibility of the deranged LFTs in up to 83% of individuals [3]. Timely identification of risk factors including age, presence of autoantibodies, and baseline LFTs, may help identify patients at risk of transaminitis. If liver enzymes are deranged during ATD treatment, a full liver screen should be performed to exclude co-existing autoimmune hepato-biliary pathologies [15,30]. As per NICE guidelines [15] and the American Thyroid Association [27], it is recommended that LFTs be monitored in all patients taking thionamides who develop clinical symptoms of liver dysfunction. American Thyroid Association (ATA) guidelines suggest stopping the medication if transaminases reach five times the upper limit of normal, and once the drug has been stopped, weekly LFT monitoring should be performed until liver enzymes have normalised [27].

## 8. Conclusions

Liver injury in individuals with GD may be multifactorial. These include direct toxicity as a result of excessive thyroid hormone production, hepatocellular anoxia, free radical production, direct action of TRAb, and use of ATD medications. In this case, the patient had a raised ALT at presentation prior to starting CBZ, which may represent direct hepatic dysfunction due to his hyperthyroid state. This followed a rapidly evolving transaminitis upon commencing CBZ. The liver function had normalised within five weeks of cessation of CBZ. The symptoms of thyrotoxicosis were managed with propranolol prior to definitive radioiodine therapy. Predictive factors such as age, high TRAb titres, tachycardia, and high fT3 levels could be used to prognosticate the development of hepatic involvement.

This case highlights the challenges of managing hyperthyroidism in the context of liver dysfunction with many possible contributing factors. Patients should be counselled regarding symptoms of hepato-biliary toxicity, including abdominal pain, pruritic, and jaundice. A multidisciplinary team can consider alternative management options such as radioiodine therapy or surgery in individuals with worsening liver dysfunction.

## 9. Key Learning Points

- Liver injury in GD may be multifactorial due to thyrotoxicosis, GD, ATDs, or other unrelated causes;
- PTU can cause hepatocellular toxicity, whereas CBZ more typically results in cholestatic jaundice without hepatic necrosis;
- LFTs should be measured before starting ATDs, with some guidelines suggesting routine LFT monitoring within 120 days of ATD initiation;
- If LFTs are >5× ULN, thionamides should be stopped.

## Figures and Tables

**Table 1:** Summary of thyroid function tests and liver function tests post-carbimazole therapy.

Time Since Presentation (Weeks)		1 Year Prior	Day 0	2	4	5	8	9	10	14	17	19	26	48	49
**Carbimazole**			**Started 40 mg od**			**Reduced to 20 mg od**	**Stopped**	**20 mg for 1 week**							
**Time since RAI** **(weeks)**										**3**	**6**	**8**	**15**	**37**	**38**
**Daily Levothyroxine Dose (mcg)**											**50**	**100**	**150**	**100**	
**Test**	**Reference ranges**														
**TSH**	0.3–4.2 IU/L (0.3–4.2 mIU/L)	1.05 (1.05)	**<0.01** **(<0.01)**	**<0.01** **(<0.01)**	**<0.01** **(<0.01)**	**<0.01** **(<0.01)**	**<0.01** **(<0.01)**	**<0.01** **(<0.01)**	**<0.01** **(<0.01)**	**<0.01** **(<0.01)**	**52.7** **(52.7)**	**24.72** **(24.72)**	**6.97** **(6.97)**	**0.14** **(0.14)**	**0.29** **(0.29)**
**fT4**	0.70–1.79 ng/dL (9–23 pmol/L)		**3.20** **(41.2)**	1.73 (22.3)	1.21 (15.6)	1.21 (15.6)	0.89 (11.4)	**2.63** **(33.9)**	**1.90** **(24.4)**	1.17 (15)	**0.44** **(5.6)**	1.06 (13.6)	1.27 (16.3)	1.33 (17.1)	1.38 (17.7)
**fT3**	155.8–389.6 pg/L (2.4–6 pmol/L)		**>1993.5** **(>30.7)**	**513** **(7.9)**		**396.1** **(6.1)**	292.2 (4.5)	**1493.5** **(23.0)**	**597.4** **(9.2)**	272.7 (4.2)	**<149.3** **(<2.3)**	194.8 (3)	188.3 (2.9)	246.8 (3.8)	
**TRAb**	0–0.9 (IU/L)		**10**												
**Bilirubin**	0–1.23 mg/dL (0–21 µmol/L)		0.99 (17)	0.99 (17)	0.82 (14)	0.64 (11)	0.88 (15)	0.94 (16)	0.82 (14)	0.76 (13)	0.64 (11)	0.88 (15)	0.64 (11)		
**ALT**	0–45 (IU/L)	30	**65**	**120**	**163**	**187**	**263**	**74**	44	32	**60**	45	**60**		
**AST**	0–40 (IU/L)				**55**		**76**	30		29	**42**	32	**42**		
**GGT**	8–60 (U/L)	24			26					20					
**ALP**	30–130 (µmol/L)	67	85	108	123	**132**	**142**	**154**	128	**160**	**152**	**149**	114		

Abnormal values are shown in bold font. Values in parenthesis are International System of Units (SI). Abbreviations: TSH, thyrotropin (thyroid-stimulating hormone); fT4, free thyroxine; fT3, free triiodothyronine; TRAb, thyrotropin receptor antibody; ALT, alanine aminotransferase; AST, aspartate aminotransferase; GGT, γ-glutamyl transferase; ALP, alkaline phosphatase.

## Data Availability

Original data generated and analysed during this study are included in this published article.
